# Crystal structure-guided design of berberine-based novel chitinase inhibitors

**DOI:** 10.1080/14756366.2020.1837123

**Published:** 2020-11-09

**Authors:** Lei Chen, Ling Zhu, Jinli Chen, Wei Chen, Xuhong Qian, Qing Yang

**Affiliations:** a School of Bioengineering, Dalian University of Technology, Dalian, China; b State Key Laboratory for Biology of Plant Diseases and Insect Pests, Institute of Plant Protection, Chinese Academy of Agricultural Sciences, Beijing, China; c Shanghai Key Laboratory of Chemical Biology, School of Pharmacy, East China University of Science and Technology, Shanghai, China; d Guangdong Laboratory for Lingnan Modern Agriculture, (Shenzhen Branch), Agricultural Genomics Institute at Shenzhen, Chinese Academy of Agricultural Sciences, Shenzhen, China

**Keywords:** Berberine, chitinase, inhibitor, structural optimisation

## Abstract

Glycoside hydrolase family 18 (GH18) chitinases play an important role in various organisms ranging from bacteria to mammals. Chitinase inhibitors have potential applications as pesticides, fungicides, and anti-asthmatics. Berberine, a plant-derived isoquinoline alkaloid, was previously reported to inhibit against various GH18 chitinases with only moderate *K*_i_ values ranging between 20 and 70 μM. In this report, we present for the first time the berberine-complexed crystal structure of *Sm*ChiB, a model GH18 chitinase from the bacterium *Serratia marcescens*. Based on the berberine-binding mode, a hydrophobic cavity-based optimisation strategy was developed to increase their inhibitory activity. A series of berberine derivatives were designed and synthesised, and their inhibitory activities against GH18 chitinases were evaluated. The compound **4c** showed 80-fold-elevated inhibitory activity against *Sm*ChiB and the human chitinase hAMCase with *K*_i_ values at the sub-micromolar level. The mechanism of improved inhibitory activities was proposed. This work provides a new strategy for developing novel chitinase inhibitors.

## 1. Introduction

Glycoside hydrolase family 18 (GH18) chitinases (EC 3.2.1.14) catalyse the degradation of chitin, a homopolymer of β-(1,4)-linked *N*-acetylglucosamine, which play important roles in various life processes. For instance, the well-studied bacterium *Serratia marcescens* produces several GH18 chitinases to efficiently degrade chitin for nutrition^1–4^. Chitinases from parasites causing nematodosis^5^ and malaria^6^ are important for the development and pathogenesis of these organisms. Insect chitinases are required to digest chitin for growth and development^7^^,^^8^. Human chitinases have been reported to be associated with asthma^9^, allergic response^10^, and other immunological disorders^11^^,^^12^. Because of the extensive roles of GH18 chitinases, inhibitors targeting these enzymes have potential applications as therapeutic agents and agrochemicals.

Over the past century, natural products have served as a source and inspiration for a large fraction of the commercial pharmaceuticals for humans, animals, and crops^13–15^. The taxonomic, functional, and chemical diversities of natural products offer inherent advantages for driving pharmaceutical discovery^13^. Many natural products have been reported to inhibit GH18 chitinases, such as allosamidin^16^, argifin^17^, argadin^18^, psammaplin A^19^, styloguanidine^20^, phlegmacin B_1_^21^, cyclo-(L-Arg-D-Pro)^22^ and methylxanthines derivatives^23^, and some of these compounds have shown practical applications.

Berberine is a plant derived isoquinoline alkaloid distributed widely in plants of the Berberidaceae, Ranunculaceae, and Papaveraceae families^24^^,^^25^. It has been used for thousands of years in traditional Chinese and Ayurvedic medicine for antimicrobial and antiprotozoal activities^24^^,^^25^. Several studies have revealed that berberine shows enormous potential in treating various diseases, including cancer, diabetes, depression, cardiovascular and hypertension^26–29^. Moreover, berberine has also been reported to have potential applications in agriculture for its antifungal, insecticidal and herbicidal activities^30–32^. Recently, it was reported to be a competitive inhibitor of GH18 chitinases, including those from the human (*Hs*Cht and hAMCase) and insect pest *Ostrinia furnacalis* (*Of*ChtI). Berberine showed moderate inhibitory activity towards *Hs*Cht, hAMCase and *Of*ChtI, with *K*_i_ values of 19, 65 and 23 μM, respectively^33^. The modest inhibitory activity of berberine against chitinases remain unsatisfactory for meeting pharmaceutical needs.

In this study, we used *Sm*ChiB, a model GH18 chitinase from the bacterium *S. marcescens*, to improve inhibitory activity of berberine. Based on the solved crystal structure of *Sm*ChiB-berberine complex, a variety of berberine analogues with much improved inhibitory activities were designed and synthesised. This work provides a new perspective for exploiting berberine as GH18 chitinase inhibitors.

## 2. Materials and methods

### 2.1. Chemicals and instruments

The uncorrected melting point (mp) was obtained with a Büchi B540 apparatus (Büchi Labortechnik AG, Switzerland). ^1^H NMR, ^13 ^C NMR and ^19 ^F NMR were recorded on a Bruker AM-400 (^1^H at 400 MHz, ^13 ^C at 100 MHz and ^19 ^F NMR at 376 MHz) spectrometer at 25 °C with samples prepared in dimethylsulphoxide (DMSO) and with tetramethylsilane (TMS) used as the internal standard. High-resolution electrospray ionisation mass spectra (HR-ESI-MS) were collected in an XEVO G2 TOF mass spectrometer (Waters, Milford, MA). The chromatographic columns for protein purification were purchased from GE Life Sciences (Beijing, China). The BCA protein assay kit was purchased from TaKaRa (Dalian, China). The yeast strain *Pichia pastoris* GS115, and the expression vectors pPIC9 and pPIC9K were purchased from Invitrogen (Beijing, China). 4-methylumbelliferyl-*β*-D-*N*,*N'*-diacetylchitobiose (MU-*β*-(GlcNAc)_2_) and berberine were purchased from Sigma (Shanghai, China). All other chemicals of the highest purity were purchased from commercial sources.

### 2.2. General procedure for the synthesis of the compounds

**Scheme 1.**
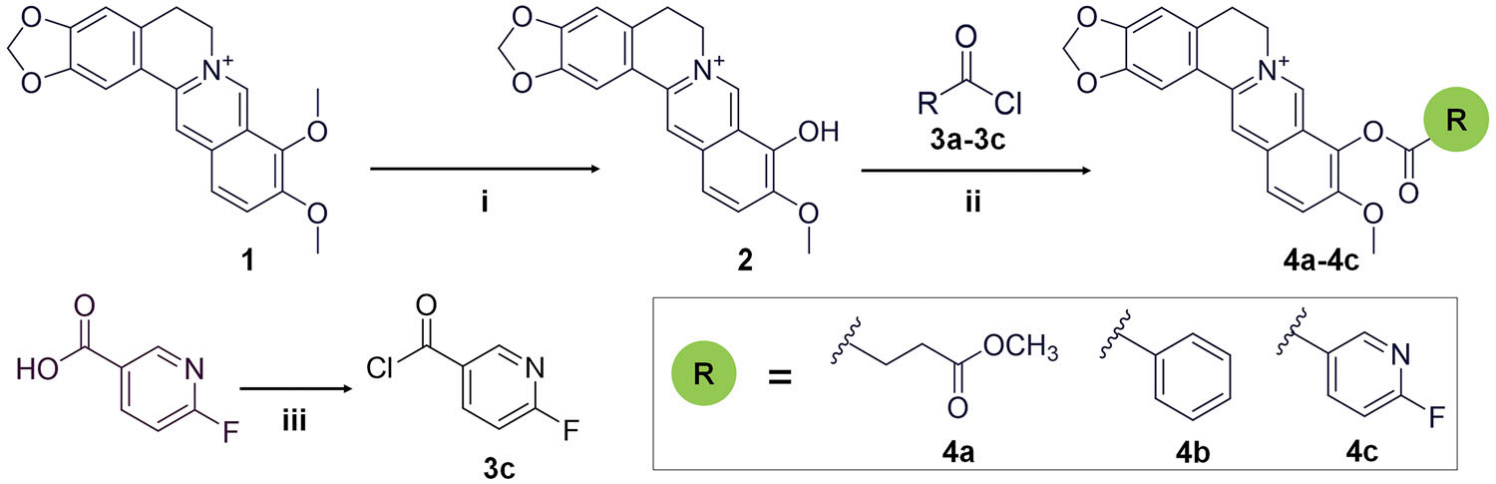
Synthetic route for the preparation of compounds **4a**-**4c** and precursors. Reagents and conditions: (i) microwave, DMF, stirred at 100 W for 1 h; (ii) acetonitrile, pyridine, stirred for 1–2 h, room temperature; (iii) oxalyl chloride, stirred for 30 min, room temperature.

The synthetic route for compounds is shown in Scheme 1. For the synthesis of the precursor berberrubine (**2**), a suspension of 1 g berberine hydrochloride (**1**) and 25 ml dimethylformamide (DMF) were stirred at 100 W for 1 h, and the solvent was removed under vacuum. The residue was purified by column chromatography (silica gel, DCM:MeOH = 3:1) to obtain the desired product berberrubine (**2**). For the synthesis of the precursor **3c**, a suspension of 141 mg (1.0 mmol) of 6-fluoronicotinic acid in 1 ml of oxalyl chloride was stirred for 30 min at room temperature. After evaporation of oxalyl chloride *in vacuo*, compound **3c** was obtained and used for the next reaction without any purification.

For the synthesis of the target compounds **4a-4c**, compounds **3a-3c** were added into a magnetically stirred solution of berberrubine (**2**) (0.20 mol) with 40 ml acetonitrile and 4 ml pyridine and stirred for 1–2 h at room temperature. The reaction was monitored by thin-layer chromatography (TLC). The resulting solid was filtered at room temperature and recrystallised twice from methyl alcohol to give the refined product.

Preparation of compound **4a**: berberrubine (**2**) was treated with methyl succinyl chloride according to the general procedure to give the desired compound **4a** as a yellow solid, yield 54.3%; mp: 192 °C (decomp). ^1^H NMR (400 MHz, DMSO-d_6_) δ 9.97 (s, 1H), 9.06 (s, 1H), 8.28 (d, *J* = 9.2 Hz, 1H), 8.22 (d, *J* = 9.2 Hz, 1H), 7.81 (s, 1H), 7.10 (s, 1H), 6.18 (s, 2H), 4.96 (t, *J* = 6.0 Hz, 2H), 4.03 (s, 3H), 3.68 (s, 3H), 3.21 (t, *J* = 6.0 Hz, 2H), 3.18 (t, *J* = 6.8 Hz, 2H), 2.81 (t, *J* = 6.8 Hz, 2H). ^13 ^C NMR (101 MHz, DMSO-d_6_) δ 172.20, 169.84, 150.45, 149.97, 147.69, 144.30, 138.12, 133.22, 132.93, 130.81, 126.82, 126.00, 121.06, 120.59, 120.31, 108.41, 105.51, 102.11, 57.24, 55.31, 51.68, 28.61, 28.52, 26.14. HR-ESI-MS calculated for C_24_H_22_NO_7_^+^ [M – Cl] ^+^: 436.1396, found: 436.1395.

Preparation of compound **4 b**: berberrubine (**2**) was treated with benzoyl chloride according to the general procedure to give the desired compound **4 b** as a yellow solid, yield 75.7%; mp: 214.5–216.2 °C. ^1^H NMR (400 MHz, DMSO-d_6_) δ 10.02 (s, 1H), 9.13 (s, 1H), 8.35 (d, *J* = 9.2 Hz, 1H), 8.31 − 8.26 (m, 3H), 7.89 − 7.82 (m, 2H), 7.71 (t, *J* = 7.8 Hz, 2H), 7.09 (s, 1H), 6.19 (s, 2H), 4.92 (t, *J* = 6.2 Hz, 2H), 4.03 (s, 3H), 3.20 (t, *J* = 6.2 Hz, 2H). ^13 ^C NMR (101 MHz, DMSO-d_6_) δ 163.39, 150.39, 149.96, 147.68, 144.47, 138.12, 134.58, 133.53, 132.96, 130.84, 130.39, 129.08, 127.95, 126.96, 125.85, 121.20, 120.65, 120.32, 108.38, 105.55, 102.11, 57.27, 55.20, 26.12. HR-ESI-MS calculated for C_26_H_20_NO_5_^+^ [M – Cl] ^+^: 426.1341, found: 426.1342.

Preparation of compound **4c**: berberrubine (**2**) was treated with 6-fluoronicotinoyl chloride according to the general procedure to give the desired compound **4c** as a yellow solid, yield 63.7%; mp:216 °C (decomp). ^1^H NMR (400 MHz, DMSO-d_6_) δ 10.06 (s, 1H), 9.16 (s, 1H), 9.10 (s, 1H), 8.89 − 8.73 (m, 1H), 8.36 (d, *J* = 9.0 Hz, 1H), 8.29 (d, *J* = 9.0 Hz, 1H), 7.84 (s, 1H), 7.56 (d, *J* = 8.2 Hz, 1H), 7.10 (s, 1H), 6.19 (s, 2H), 4.90 (t, *J* = 4.1 Hz, 2H), 4.05 (s, 3H), 3.21 (t, *J* = 0.5 Hz, 2H). ^13 ^C NMR (101 MHz, DMSO-d_6_) δ 166.30 (d, *J* = 244.4 Hz), 161.78, 151.38 (d, *J* = 17.1 Hz), 150.89, 150.53, 148.22, 144.98, 144.85 (d, *J* = 9.5 Hz), 138.79, 133.49, 133.31, 131.37, 127.79, 126.42, 123.59 (d, *J* = 4.4 Hz), 121.50, 121.12, 120.80, 111.08 (d, *J* = 37.9 Hz), 108.91, 106.04, 102.63, 57.85, 55.80, 26.63. ^19 ^F NMR (376 MHz, DMSO-d_6_) δ −60.58 (d, *J* = 7.7 Hz, 1 F). HR-ESI-MS calculated for C_25_H_18_FN_2_O_5_^+^ [M – Cl] ^+^: 445.1200, found: 445.1198.

### 2.3. Enzyme preparation

ChiB from *S. marcescens* was expressed in *Escherichia coli* BL21 (DE3). The other GH18 chitinases including the catalytic domains of *Of*ChtI from *O. furnacalis*, human *Hs*Cht and human hAMCase were expressed in *P. pastoris* GS115. All the proteins were purified by immobilised metal affinity chromatography (IMAC) as described previously^34^. The purities of the target proteins were analysed by SDS-PAGE followed by Coomassie Brilliant Blue R-250 staining.

### 2.4. Inhibitory activity assay

The activity of GH18 chitinases were determined using MU-(GlcNAc)_2_ as a substrate. The reaction mixtures used for determining the inhibitor activity of compounds consisted of 100 μL of 20 nM enzyme, 4 μM MU-(GlcNAc)_2_, 1 or 10 μM inhibitors and 2% DMSO in the buffer (20 mM sodium phosphate, pH 6.0, for *Sm*ChiB, *Of*ChtI and *Hs*Cht; 20 mM sodium citrate, pH 5.2, for hAMCase). The reaction in the absence of compounds was used as a positive control. After incubating at 30 °C for 25 min, an equal volume of 0.5 M sodium carbonate was added to the reaction mixtures to terminate the reaction, and the fluorescence produced by the released MU was quantified using a Varioskan Flash microplate reader (Thermo Fisher Scientific, Waltham, MA), with excitation and emission wavelengths of 360 and 450 nm, respectively. Experiments were performed in triplicate. For *K*_i_ value determination, three substrate concentrations (4, 8, and 12 μM for *Sm*ChiB, *Hs*Cht and hAMCase, 1, 2, and 4 μM for *Of*ChtI) and varied inhibitor concentrations were used. The *K*_i_ values and types of inhibition were determined by linear fitting of the data in Dixon plots.

### 2.5. Fluorescence measurements

Tryptophan fluorescence (295 nm excitation) was measured at 25 °C from 300 to 400 nm with a Varioskan Flash microplate reader using excitation and emission band passes of 5 nm. Fluorescence quenching experiments were performed in a 200 μL mixture containing 1 μM protein in the buffer (20 mM sodium phosphate, pH 6.0, for *Sm*ChiB, *Of*ChtI and *Hs*Cht; 20 mM sodium citrate, pH 5.2, for hAMCase), and by the successive addition of aliquots of compounds stock solution. Since the crystal structure confirmed that berberine binding to *Sm*ChiB in a 1:1 ratio, the dissociation constant *K*_d_ were then determined using the modified Stern-Volmer equation^35^^,^^36^.

$$F_{0}/(F_{0}-F)\;=\;1/f_{a}+K_{d}/f_{a}[Q]$$

Where, *F*_0_ and *F* are fluorescence intensities in the absence and presence of compounds, *f*_a_ is the fraction of the accessible fluorescence, [*Q*] is the concentration of the compounds and *K*_d_ is the dissociation equilibrium constant.

### 2.6. Protein crystallisation and structure determination

Crystallisation experiments were performed by the hanging drop–vapor diffusion method at 4 °C. *Sm*ChiB was firstly desalted in the 20 mM Tris-HCl with 50 mM NaCl, pH 8.0, and concentrated to 10 mg/mL before crystallisation. The crystals of free *Sm*ChiB were obtained in 1.0–2.0 M ammonium sulphate, 10–20% glycerol, 100 mM HEPES, pH 7.0 and then soaked with berberine to a final concentration of 1 mM for 1 h to yield the *Sm*ChiB-berberine complex. The crystals were cryoprotected by immersion in mother liquor containing 25% glycerol and flashed-cooled in liquid nitrogen.

X-ray diffraction data of the complex were collected at the National Centre for Protein Science, Shanghai (BL18U, Pilatus 3–6 M detector). The structure of *Sm*ChiB complexed with berberine was solved by molecular replacement with PHASER^37^ using the structure of free *Sm*ChiB (PDB: le6n) as the search model. The PHENIX^38^ was used for structure refinement. Coot^39^ was used for manually building and extending the molecular models. PROCHECK^40^ was used to check the stereochemical quality of the models. The coordinates of the *Sm*ChiB-berberine complex were deposited in the Protein Data Bank (PDB) as entries 7C34. All structure figures were generated using PyMOL (DeLano Scientific LLC).

### 2.7. Molecular docking

The PDB files of berberine and its analogs were prepared using PRODRG^41^. The crystal structures of *Sm*ChiB (PDB: 7C34), *Of*ChtI (PDB: 3WQW), *Hs*Cht (PDB: 1HKK) and hAMCase (PDB: 2YBT) were prepared by PyMOL as the templates for the molecular docking. MGLTools was used to generate the PDBQT files of the proteins and compounds. The active site boxes were set at 50 × 50 × 50 Å^3^, 70 × 70 × 60 Å^3^, 70 × 70 × 70 Å^3^ and 60 × 80 × 60 Å^3^ for *Sm*ChiB, *Hs*Cht, *Of*ChtI and hAMCase using AutoGrid4^42^, respectively. Molecular Docking were performed by AutoDock4^42^ using the Lamarckian genetic algorithm with a population size of 100 individuals, 25000000 energy evaluations, and 27000 generations. Plausible docking models were selected from the abundant clusters [root-mean-square deviation (RMSD) = 2 Å] that had the lowest binding energies.

## 3. Results and discussion

### 3.1. The binding mode of berberine in SmChiB

Berberine was reported as a competitive inhibitor towards several GH18 chitinases including *Of*ChtI, *Hs*Cht, and hAMCase^33^. In this study, we found berberine was also a competitive inhibitor against *Sm*ChiB, a well-studied GH18 chitinase, with a *K*_i_ value of 11.79 μΜ (Table 1, Figure S1). To improve inhibitory activity, the binding mode of berberine in the *Sm*ChiB active pocket was analysed. The crystal structure of the *Sm*ChiB-berberine complex was obtained by soaking and was resolved to a resolution of 1.94 Å. The statistics of data collection and structure refinement are shown in Table 2. The coordinates of the *Sm*ChiB–berberine complex have been deposited in the PDB under accession number 7C34.

**Table 1.** Inhibitory activities and binding affinities of the compounds towards different GH18 chitinases.

	μM							
	*Sm*ChiB		*Of*ChtI		*Hs*Cht		hAMCase	
Compounds	*K*_i_	*K*_d_	*K*_i_	*K*_d_	*K*_i_	*K*_d_	*K*_i_	*K*_d_
Berberine	11.79	11.98	23^33^	20.61	19^33^	21.89	65^33^	58.03
**4a**	0.68	1.12	6.43	–	8.23	–	7.43	–
**4b**	2.36	2.99	7.28	–	11.39	–	4.62	–
**4c**	0.15	0.19	3.03	2.58	0.35	0.34	0.80	1.12

**Table 2.** Details of data collection and structure refinement.

	*Sm*ChiB-Berberine
Space group	*P* 2_1_ 2_1_ 2_1_
Unit-cell parameters	
* a*, *b*, *c* (Å)	56.09, 103.77, 186.51
* α*, *β*, *γ* (°)	90, 90, 90
Wavelength (Å)	0.9778
Temperature (K)	100
Resolution (Å)	29.78-1.94 (2.008-1.94)
Unique reflections	81619 (7997)
Observed reflections	991829
* R*_merge_	0.05 (0.152)
Average multiplicity	7.0 (6.8)
Mean *I/σI*	8.55 (2.1)
Completeness (%)	99.87 (99.27)
R/R_free_	0.163/0.195
Protein residues	992
Water molecules	740
Other atoms	84
Root mean square deviations	
Bond lengths (Å)	0.012
Bond angles (°)	1.38
Wilson B factor (Å^2^)	27.44
Average B factor (Å^2^)	32.24
Protein atoms	31.30
Water molecular	33.40
Ligand molecules	80.50
Ramachandran plot (%)	
Most favoured	98
Additionally allowed	2
Outliers	0
PDB code	7C34

**Figure 1.**
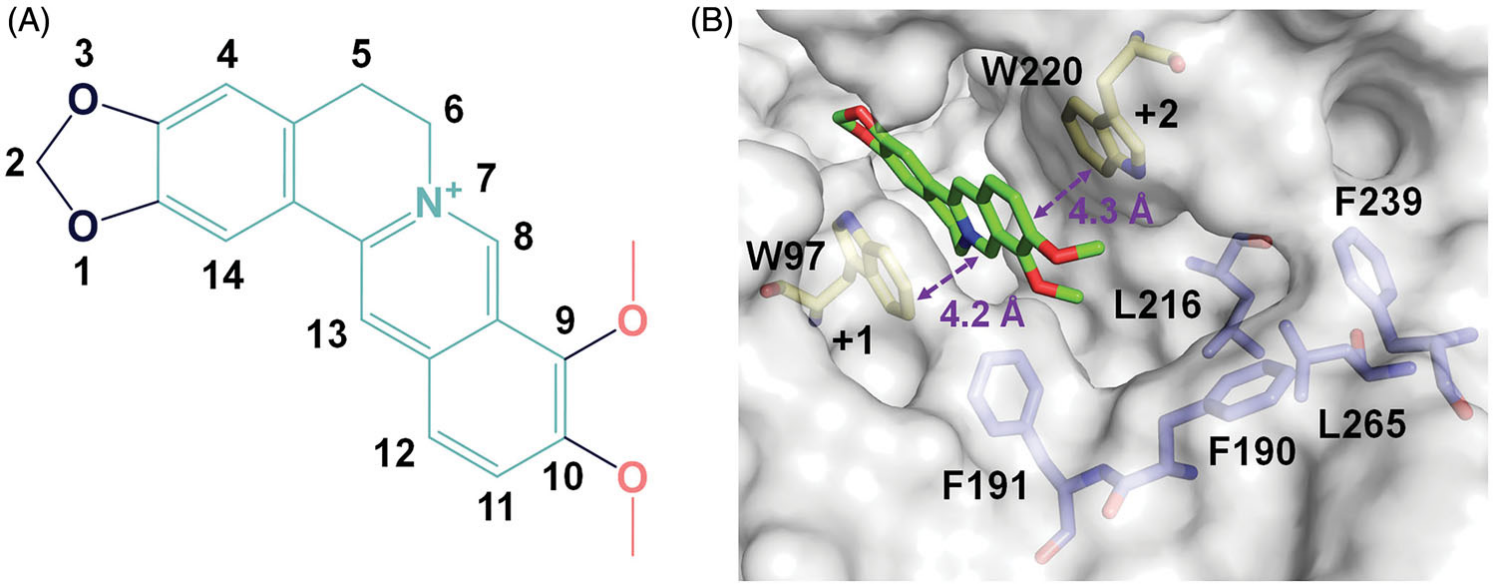
Crystal structure of *Sm*ChiB in complex with berberine. (A) Structure of berberine. The conjugate tetracycle plane is shown in green-cyan, the 9-*O*-methoxy and 10-*O*-methoxy moieties are shown in pink. (B) Binding mode of berberine in the active pocket of *Sm*ChiB. Berberine is shown in stick representation with carbon atoms in green. The aromatic residues that stack with berberine are labelled and shown in stick representation with carbon atoms in yellow. The amino residues forming the hydrophobic cavity extended near the +2 subsite are labelled and shown as stick representation with carbon atoms in blue. The numbers indicate the subsite to which the berberine is bound.

The crystal complex structure clearly revealed the location of berberine in the active pocket of *Sm*ChiB (Figure 1(B)). Berberine occupied the active pocket across the sugar-binding subsites +1 and +2 which was characterised by the aromatic Trp^97^ and Trp^220^, respectively. The nomenclature for sugar-binding subsites was proposed by Davies et al^43^, where subsite + n represents the reducing end and subsite –n represents the non-reducing end. The conjugate tetracycle plane (Figure 1(A)) of berberine was sandwiched by Trp^97^ and Trp^220^ to form π-π stacking interactions within distance of 4.2 and 4.3 Å respectively. This observation was consistent with our previous work that berberine inhibited GH18 chitinases mainly via π-π stacking interactions between the tetracycle plane and the aromatic residues in the binding pockets^33^. This finding inspired us to retain the conjugate plane of berberine in the next design of berberine-based inhibitors. Moreover, an unoccupied hydrophobic cavity formed by residues Phe^190^, Phe^191^, Leu^216^, Phe^239^, and Leu^265^ was identified (Figure 1(B)). This space provides a favourable opportunity for the optimisation of berberine at its 9-*O*-position or 10-*O*-position. As the 9-*O*-position had been reported to be readily modified^44–46^, it might be a candidate site for derivation of berberine for improving inhibitory activity.

### 3.2. Design, synthesis and biological activity of berberine analogs

Modification of berberine was performed by substituting a variety of groups at the 9-*O*-position. Three hydrophobic and bulky substituents including a methyl succinyl group, benzoyl group and 6-fluoronicotinoyl group were selected. The addition of a carbonyl group, fluorine atom and nitrogen atom in the substituents were expected to form hydrogen bonds with surrounding residues in the hydrophobic cavity. The compounds **4a**-**4c** were obtained according to the synthetic route outlined in Scheme 1. Briefly, the key intermediate berberrubine (**2**) was obtained via microwave reaction^47^. Berberrubine was then treated with excess acyl chloride **3a-3c** in anhydrous acetonitrile (pyridine as catalyst) to produce the crude target compounds **4a-4c**. The crude compounds were filtered at room temperature and then recrystallized twice from methyl alcohol to give the refined product. The reagents and materials that were used in the syntheses are easily commercially available, and the synthetic route resulted in high atom economy with good utilisation of reactant atoms in the end products.

The newly synthesised compounds **4a-4c** were then evaluated for inhibition activities against *Sm*ChiB. The results revealed that all three compounds were competitive inhibitors against *Sm*ChiB and showed improved inhibitory activities when compared with that of berberine. The *K*_i_ values of compound **4a-4c** against *Sm*ChiB were 0.68, 2.36, and 0.15 μM, respectively (Table 1, Figure S1).

Berberine have been reported to be a broad-spectrum inhibitor of several GH18 chitinases, and therefore the inhibitory activities of compounds **4a-4c** against other GH18 chitinases, including *Of*ChtI, *Hs*Cht, and hAMCase, were also evaluated. As shown in Table 1 and Figure S2, compounds **4a-4c** exhibited various degree of improvement in inhibitory activities against *Of*ChtI, *Hs*Cht, and hAMCase when compared to berberine. In particular, compound **4c** showed the highest inhibitory activity against all tested GH18 chitinases. The *K*_i_ values of compound **4c** towards *Of*ChtI, *Hs*Cht, and hAMCase were 3.03, 0.35, and 0.80 μM, respectively.

### 3.3. Inhibition mechanism of the compounds

According to our optimisation strategy, the modification of 9-*O*-position may improve the inhibitory activity of berberine by enhancing its binding affinities towards chitinases. To confirm this hypothesis, *K*_d_ values were determined using tryptophan fluorescence quenching experiments. As revealed in Table 1 and Figure S3, berberine binds to *Sm*ChiB with a *K*_d_ of 11.98 μM. The *K*_d_ values of compounds **4a-4c** against *Sm*ChiB were 1.12, 2.99 and 0.19 μM, respectively, showing good agreement with *K*_i_ values (Table 1 and Figure S1). The *K*_d_ values of berberine towards *Of*ChtI, *Hs*Cht and hAMCase were 20.61, 21.89 and 58.03 μM, respectively, which were also consistent with the *K*_i_ values. Compared with berberine, compound **4c** showed a much lower *K*_d_ against *Of*ChtI, *Hs*Cht and hAMCase with values of 2.58, 0.34 and 1.12 μM (Table 1, Figure S4). These results showed that modification at the 9-*O*-position increased binding affinity.

**Figure 2.**
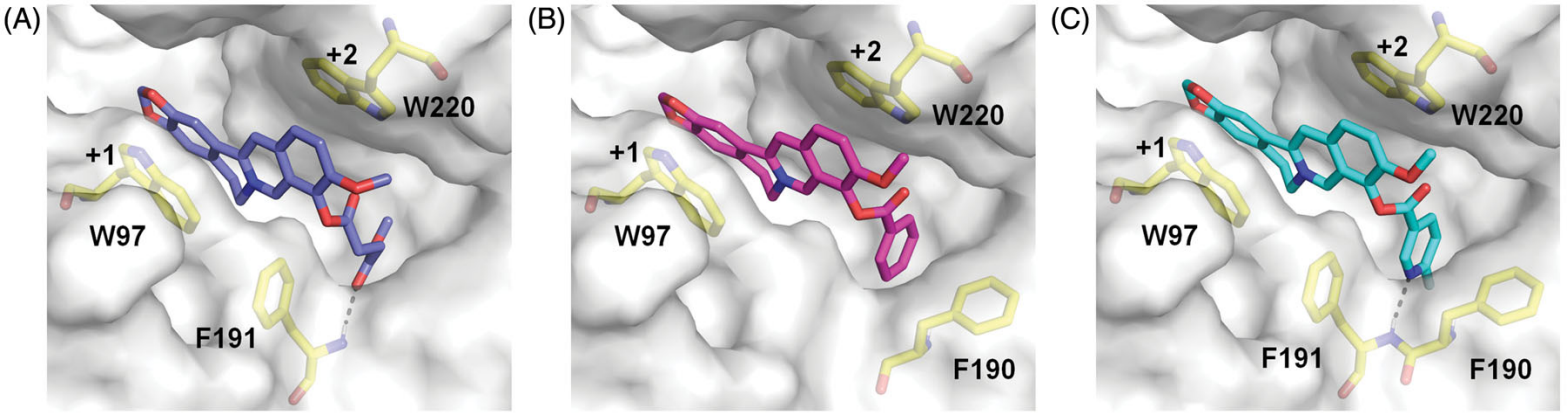
Modelled complex structures of compounds **4a**-**4c** in *Sm*ChiB. Details of the interaction of compound **4a** (A), **4 b** (B) and **4c** (C) with *Sm*ChiB. Compound **4a** is shown in blue, compound **4 b** is shown in magenta and compound **4c** is shown in cyan. Residues that participate in binding are shown in yellow. Hydrogen bonds are shown as dashed black lines. The numbers indicate the subsite to which the compounds are bound.

To further understand the inhibitory mechanism of compounds **4a-4c**, the structure-based molecular docking was performed to reveal the details responsible for the enhanced binding affinity. The binding modes of **4a-4c** against *Sm*ChiB were nearly identical to that of berberine. The tetracycle moiety of the compounds occupied the substrate-binding cleft from subsites +1 to +2 via π–π stacking interactions with Trp^97^ and Trp^220^. The 9-*O*-position substituents of the berberine analogues inserted into the extended hydrophobic pocket (Figure 2). As a result, the berberine analogues were able to form additional interactions such as hydrogen bonds and stacking interactions with surrounding residues of *Sm*ChiB, which may account for their increased inhibitory activities. For example, compound **4a** formed a hydrogen bond with Phe^191^ via the carbonyl group of the methyl succinyl substituent. Compound **4 b** formed a T-shaped stacking interaction with Phe^190^ via its benzene ring. For compound **4c**, the 6-fluoronicotinoyl substituent group formed both hydrogen bond and T-shaped stacking interactions with Phe^191^ and Phe^190^, respectively, which may account for its optimal inhibitory activity against *Sm*ChiB.

**Figure 3.**
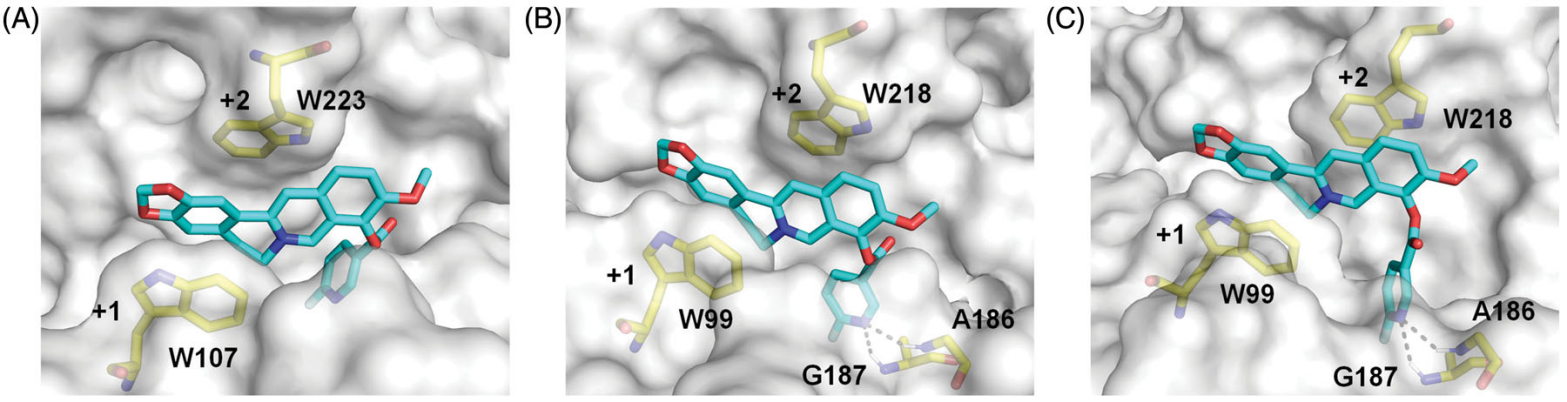
Modelled structures of compound **4c** in *Of*ChtI (A), *Hs*Cht (B), and hAMCase (C). Compound **4c** is shown in stick representation with carbon atoms in cyan. Amino acids that interact with compound **4c** are labelled and shown in stick representation with carbon atoms in yellow. Hydrogen bonds are shown as dashed black lines. The numbers indicate the subsite to which compound **4c** is bound.

As compound **4c** showed the highest inhibitory activity against all GH18 chitinases studied, its binding mechanism towards *Of*ChtI, *Hs*Cht and hAMCase were also studied by molecular docking (Figure 3). The molecular docking results revealed that compound **4c** inhibited these enzymes via a similar mechanism. Compound **4c** bound in the active pocket across the +1 and +2 subsites of the enzymes. The conjugate tetracycle plane formed π–π stacking interactions with conserved tryptophan residues (Trp^107^ and Trp^223^ in *Of*ChtI, Trp^99^ and Trp^218^ in *Hs*Cht and hAMCase). The 6-fluoronicotinoyl group inserted into the extended hydrophobic pockets of enzymes that results in hydrogen bonds formation between the nitrogen atom of the heterocyclic aromatic ring and the hydrogen atom of surrounding residues (Gly^187^ and Ala^186^ in *Hs*Cht and hAMCase). These hydrogen bonds appear to be important for its inhibitory activity, because the *K*_i_ values of compound **4c** against *Hs*Cht and hAMCase were 54 and 81-fold higher than that of berberine, whereas the failure formation of hydrogen bond of compound **4c** with *Of*ChtI and the inhibitory activity only increased 7.5-fold higher than that of berberine (Table 1, Figure S2).

## 4. Conclusions

In the current study, we successfully developed a structure-optimisation strategy of berberine to improve the inhibitory activity against GH18 chitinases by taking advantage of an unoccupied hydrophobic cavity of the target enzyme. This work firstly reveals the binding mode of the natural product berberine in GH18 chitinase and provides a new path for exploring berberine-based molecules as promising GH18 chitinase inhibitors.

## Supplementary Material

Supplemental Material
