# Supplementary material for: Crystal structure-guided design of berberine-based novel chitinase inhibitors
Source: J Enzyme Inhib Med Chem. 2020 Nov 9;35(1):1937–43. doi: 10.1080/14756366.2020.1837123 (PMC7655067; doi:10.1080/14756366.2020.1837123)
Supplement: Supplemental Material [file IENZ_A_1837123_SM7153.pdf]

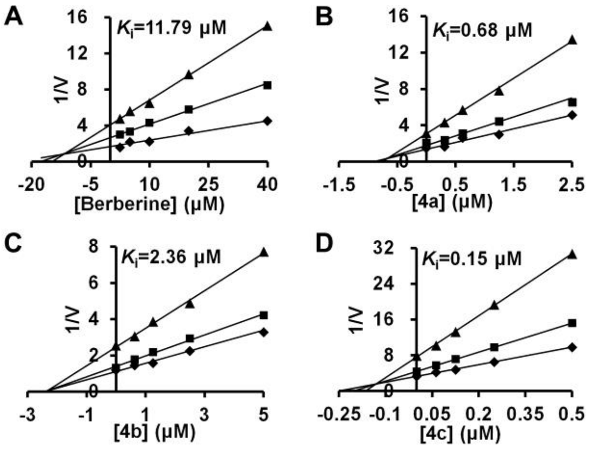

Figure S1. Inhibition kinetics of berberine and its analogues toward *Sm*ChiB. (A) Inhibition kinetics of berberine toward *Sm*ChiB; (B) Inhibition kinetics of **4a** toward *Sm*ChiB; (C) Inhibition kinetics of **4b** against *Sm*ChiB; (D) Inhibition kinetics of **4c** against *Sm*ChiB.

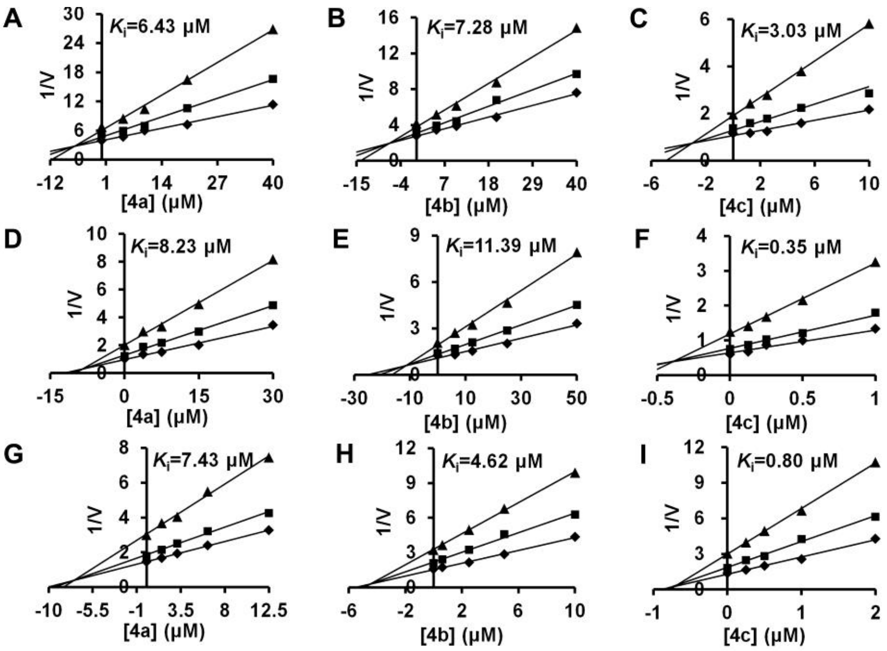

Figure S2. Inhibition kinetics of berberine analogues toward GH18 chitinases. (A), (D) and (G), inhibition kinetics of **4a** toward *Of*ChtI, *Hs*Cht, and hAMCase; (B), (E) and (H), inhibition kinetics of **4b** toward *Of*ChtI, *Hs*Cht, and hAMCase; (C), (F) and (I), inhibition kinetics of **4c** against *Of*ChtI, *Hs*Cht, and hAMCase.

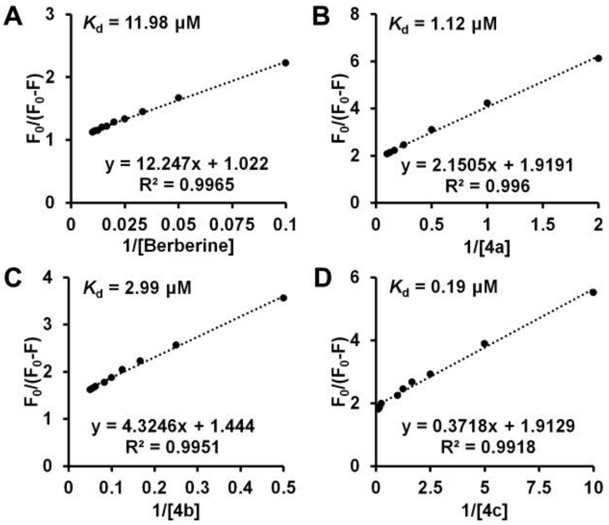

Figure S3. Tryptophan emission quenching of *Sm*ChiB by berberine and its analogues. The modified Stern-Volmer plots for fluorescence quenching of *Sm*ChiB by berberine (A), **4a** (B), **4b** (C) and **4c** (D).

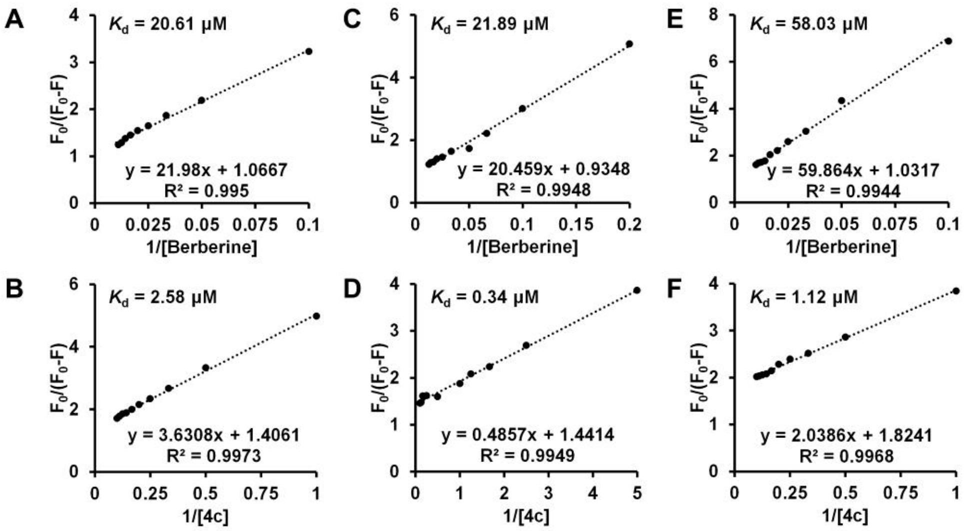

Figure S4. Tryptophan emission quenching of GH18 chitinases by berberine and analogue **4c**. (A), (C) and (E), the modified Stern-Volmer plots for fluorescence quenching of *Of*ChtI, *Hs*Cht and hAMCase by berberine, respectively; (B), (D) and (F), the modified Stern-Volmer plots for fluorescence quenching of *Of*ChtI, *Hs*Cht and hAMCase by **4c**, respectively.
